# Adductor focal laryngeal Dystonia: correlation between clinicians’ ratings and subjects’ perception of Dysphonia

**DOI:** 10.1186/s40734-017-0066-y

**Published:** 2017-12-13

**Authors:** Celia Faye Stewart, Catherine F. Sinclair, Irene F. Kling, Beverly E. Diamond, Andrew Blitzer

**Affiliations:** 1 New York University, Steinhardt School of Culture, Education, and Human Development, 665 Broadway, Suite 900, New York, NY 10012 USA; 2 Icahn School of Medicine at Mount Sinai, 425 West 59th Street, New York, NY 10019 USA; 3 Mannes College the New School for Music, 55 West 13th St, New York, 10011 USA; 4 Adelphi University, 75 Varick St, New York, 10013 USA; 5 Clinical Endocrinology and Metabolism, Endocrine Society, 2055 L Street NW, Suite 600, Washington, DC, 20036 USA; 6 Columbia University College of Physicians and Surgeons, Neurology, Icahn School of Medicine at Mt. Sinai, Center for Voice and Swallowing Disorders, 425 West 59th Street, New York, NY 10019 USA

**Keywords:** Dystonia, Voice, Quality of life, Botulinum toxin, Adductor focal laryngeal dystonia

## Abstract

**Background:**

Although considerable research has focused on the etiology and symptomology of adductor focal laryngeal dystonia (AD-FLD), little is known about the correlation between clinicians’ ratings and patients’ perception of this voice disturbance. This study has five objectives: first, to determine if there is a relationship between subjects’ symptom-severity and its impact on their quality of life; to compare clinicians’ ratings with subjects’ perception of the individual characteristics and severity of AD-FLD; to document the subjects’ perception of changes in dysphonia since diagnosis; to record the frequency of voice arrest during connected speech; and, finally, to calculate inter-clinician reliability based on results from the Unified Spasmodic Dysphonia Rating Scale (USDRS) (Stewart et al, J Voice 1195-10, 1997).

**Methods:**

Sixty subjects with AD-FLD who were receiving ongoing injections of BoNT participated in this study. Subjects’ mean age was 60.78 years and their mean duration of symptoms was 16.1 years. Subjects completed the Disease Symptom Questionnaire (DSQ) (specifically designed for this study) and the Voice Handicap Index-10 (VHI-10) (Jacobson et al, Am J Speech Lang Pathol 6:66–70, 1997) to measure the symptoms of their dysphonia and the impact of the disease on their quality of life.

Two speech-language pathologists and two laryngologists used the Voice Arrest Measure (VAM) (specifically designed for this study) and the USDRS to independently rate voice recordings of 56/60 subjects.

**Results:**

The mean VHI-10 score was 21.3 which is clinically significant. The results of the DSQ and the USDRS were highly correlated. The most severe symptoms identified by both subjects and clinicians were roughness, strain-strangled voice quality, and increased expiratory effort. Voice arrest, aphonia, and tremor were uncommon. Subjects rated their current voice quality at the time of reinjection (i.e., at the time of the study) as significantly better than at the time of their initial AD-FLD diagnosis (*p* < 0.0001). Inter-clinician reliability on the USDRS was significant at the 0.001 level.

**Conclusions:**

The findings from the VHI-10 suggest that AD-FLD has a profound impact on quality of life. The results of the DSQ and the USDRS suggest that there is a strong correlation between subjects’ perception and clinicians’ assessment of the individual symptoms and the severity of the dysphonia. The findings from the VAM suggest that voice arrests are infrequent in subjects with AD-FLD who are receiving ongoing BoNT injections. The strong inter-clinician reliability on the USDRS suggests that it is an appropriate measure for identifying symptoms and severity of AD-FLD.

**Electronic supplementary material:**

The online version of this article (10.1186/s40734-017-0066-y) contains supplementary material, which is available to authorized users.

## Background

Patients with AD-FLD whose management involves ongoing injections of BoNT schedule reinjection based on the reemergence of their symptoms. [1] In addition to the patient’s report of symptoms, clinicians rely on medical history, visualization of the vocal folds, and the vocal phenomena associated with AD-FLD (e.g., strained-strangled voice quality, roughness, and increased expiratory effort) to determine the severity of the dysphonia and make treatment decisions. [2–4] The reliability of perceptual ratings between laryngologists and speech-language pathologists, however, is not well documented. Further, the literature related to AD-FLD has not compared clinicians’ ratings and subjects’ perception of these voice symptoms.

It is well accepted that voice problems may impede the development of social relationships as well as negatively affect educational, and vocational growth [5–10]. Although the VHI-10 [5] has been used to measure the impact of AD-FLD on quality of life, it does not chronicle the patient’s perception of specific vocal symptoms or the severity of the dysphonia associated with this complex, neurological disorder. [5–8] To this end, the investigators developed The Disease Symptom Questionnaire (DSQ) (see Additional file 1), a self-rating instrument on which subjects identify the specific symptoms associated with AD-FLD and rate their severity. We can then examine the relationship between patients’ perception documented on the DSQ with clinicians’ ratings from the USDRS [11].

Historically, voice arrest has been described as a central phenomenon and a necessary component in the diagnosis of AD-FLD. [12–15] Nevertheless, in our clinical experience voice arrest has been relatively infrequent. The Voice Arrest Measure (VAM) was developed to document the frequency and duration of voice arrest during connected speech.

As is the case with many medications, BoNT washes out of the system over time requiring ongoing re-injection in order to maintain the patient’s improvement in voice production. The expected duration of benefit from injections is three to four months, with subjects requiring ongoing reinjections to maintain an easy, efficient manner of phonation. [16–20] The patient determines when it is time to seek reinjection, but the criteria for their decision remain unclear. Thus far, no studies have compared the subjects’ rating of symptoms at baseline with those ratings just prior to reinjection to determine whether BoNT treatment may alter the disease process.

This study has five aims: first, to ascertain the relationship between the VHI-10 and the DSQ; to compare clinicians’ ratings from the USDRS to the subjects’ perceptual ratings from the DSQ; to identify the subjects’ perception of changes in their dysphonia since diagnosis as measured by the DSQ; to determine the frequency of voice arrests based on the (VAM); and, finally, to measure the inter-clinician (i.e., two laryngologists and two speech-language pathologists) reliability from the ratings on the USDRS.

## Methods

### Subjects

Between March 20 and July 17, 2012, sixty adults (forty-two females 70% and 18 males 30%) suffering from AD-FLD presented to a single clinical research center for continuing treatment with injections of BoNT. Subjects mean age was 60.78 years (SD 14.13) and the mean time between diagnosis of AD-FLD and participation in the study was 16.8 years (SD 9) with a mean age at the onset of symptoms of 46.05 years (SD 14.28). Data were collected just prior to reinjection when subjects self-determined that the symptoms had deteriorated to the point when reinjection was necessary. The mean interval between the previous injection of BoNT and participation in the study was 20 weeks (SD 12.04), with 49/52 (94%) having received between five and forty injections.

The inclusion criteria for this study were based on a previous diagnosis of AD-FLD, participation in ongoing laryngeal injections of botulinum toxin (BoNT) to minimize symptoms, and the subjects reported benefit from BoNT injections. Subjects had self-selected to return for continuing BoNT management of their voice symptoms when the benefits of the BoNT injections had diminished. Exclusion criteria included co-existing upper respiratory tract infection, concomitant neurological disorders, and non-neurological laryngeal pathologies. Although 100% of the subjects were known to the laryngologists, only 5% of the subjects were known to the speech-language pathologists.

The subjects gave informed consent to participate in a video recording of a voice assessment and to complete self-evaluation questionnaires (Voice Handicap Index-10^4^ and Disease Symptom Questionnaire) as part of the study. This study was approved by the Institutional Review Board (IRB) at New York University School of Medicine.

### Self-rating scales

#### VHI-10

The Voice Handicap Index-10 (VHI-10) [5] measures the impact of the voice disorder on the patients’ quality of life.

#### DSQ

The Disease Symptom Questionnaire (DSQ) (see Additional file 1) was designed as part of this study to ascertain the subjects’ perception of the individual characteristics and severity of the voice problem at the time of initial diagnosis and at the time of the study. Subjects were asked to document their current age, medical comorbidities, age at diagnosis, and time since initial diagnosis, and since last injection. The authors designed the DSQ to parallel the symptoms rated on the Unified Spasmodic Dysphonia Rating Scale (USDRS) ensuring that the subjects would rate the same symptoms as the otolaryngologists and speech-language pathologists (e.g., severity of dysphonia, hoarse-rough-husky voice quality, strain-strangled, increased expiratory effort).

The DSQ rates the severity of the current symptoms and the symptoms at diagnosis using a 5-point interval scale (i.e., mild, mild-moderate, moderate, moderate-severe or severe). One question asks subjects to compare the severity of their symptoms at diagnosis to their symptoms at the time of participation in the study using a 3-point interval scale (i.e., less severe than before treatment, the same as before treatment, and worse than before treatment).

### Clinician-rating scales

#### USDRS

The Unified Spasmodic Dysphonia Rating Scale (USDRS) [11] provides a framework for clinicians to rate the individual characteristics and the severity of a subject’s dysphonia using a 7-point Likert scale.

#### VAM

The Voice Arrest Measure (VAM) (see Additional file 2) was designed as part of this study for clinicians to identify the occurrence and duration of each voice arrest during the oral reading of the first paragraph of the Rainbow Passage. [21] This scale is based on the Stuttering Severity Index (SSI). [22] The duration of each voice arrest identified on the VAM ranges from momentary (< 1 s), brief (1–2 s), or long (>2 s in duration).

### Procedures

A student intern recorded voice samples in a quiet room on a Kay/Pentax VLS 1190 STK distal chip camera system with an Audio-Technica Pro 8HEmW head-mounted microphone placed 4 in. from the subjects’ mouth. The subjects read the first paragraph of the Rainbow Passage aloud, described the Cookie Theft picture [23], and counted from eighty to eighty-nine. Two laryngologists and two speech-language-pathologists independently reviewed the video/audio recordings in a quiet room and rated the severity of the voice symptoms on the USDRS. The mean of the severity of dysphonia rated by the four clinicians was calculated and compared to each subject’s ratings on the DSQ. The clinicians reviewed the Rainbow Passage a second time and used the VAM to identify the occurrence and duration of voice arrest.

Information pertaining to age at data collection and symptom onset, number of years since initial diagnosis of AD-FLD, time since first BoNT injection, number of BoNT injections, and time since most recent BoNT injection was obtained on the DSQ and analyzed with descriptive statistics.

Inter-clinician reliability for the severity of dysphonia and individual voice symptoms evaluated on the USDRS was calculated using SPSS version 23 interclass correlation coefficient. Paired sample T-tests were used to compare the subject’s ratings (DSQ) of the severity of symptoms with the clinicians’ ratings (USDRS). The relationship between the DSQ and the USDRS was calculated with a two-tailed correlation. Due to the comparative infrequency of voice arrest identified by the clinicians on the VAM, only descriptive statistics were used to describe these data.

## Results

### The impact of AD-FLD on quality of life

The mean VHI-10 score was 21.3 (+/−9.6) with 86.7% of subjects scoring greater than 11, which indicates a voice handicap that substantially affects quality of life. Subjects identified symptoms across all three domains included on the VHI-10 (i.e., functional, physical, and emotional). The VHI-10 scores were positively correlated with the overall severity of dysphonia on the DSQ and negatively correlated with age at the time of diagnosis such that those subjects who were younger at the time of diagnosis reported higher VHI-10 scores. The VHI-10 was not correlated with number of injections, time since diagnosis, or time since previous BoNT injection. See Table 1.

**Table 1:** Correlation of VHI-10 with DSQ *N* = 60

	VHI-10	
	Pearson Correlation	Significance
Overall Severity of Dysphonia on the DSQ	0.536	.000*
Age at Time of Diagnosis	−0.328	.011*
Number of Injections	10.162	.25
Time Since Diagnosis	−0.049	.71
Duration Since Previous BoNT Injection	−0.09	.94

^*^Finding was statistically significant at the .01 level

### Correlations between the Subject’s ratings of the current severity of the Dysphonia and the individual voice symptoms

Pearson analysis revealed a strong positive correlation (see Table 2) between the subjects’ ratings on the DSQ of severity of dysphonia and the individual symptoms of strain-strangled, roughness, voice tremor, and voice arrest.

**Table 2:** Correlations Between the Subjects’ Ratings of the Current Severity of the Dysphonia and the Individual Voice Symptoms *N* = 56

	Subjects’ Ratings of Severity of Dysphonia	
	Pearson Correlation	Significance (2-tailed)
Subjects’ Ratings Strain-Strangled	0.673	0.000*
Subjects’ Ratings Roughness	0.549	0.000*
Subjects’ Ratings Increased Expiratory Effort	0.187	0.000*
Subjects’ Ratings Voice Tremor	0.477	0.000*
Subjects’ Ratings Voice Arrest	0.564	0.000*

^*^All findings were statistically significant at the 0.01 level

### Relationship between the VHI-10 and the DSQ

Correlations between subjects’ ratings on the VHI-10 and the individual DSQ items were significant for severity of dysphonia, strain-strangled voice quality, roughness, voice tremor, and voice arrest (see Table 3). These positive correlations suggest that more severe voice (e.g., roughness) and sensory (e.g., increased expiratory effort) symptoms are associated with greater handicap as assessed on the VHI-10.

**Table 3:** Correlations Between Ratings on the VHI-10 and Severity of Current Symptoms on DSQ N = 56

	Subjects’ Ratings on the VHI-10	
	Pearson Correlation	Significance (2-tailed)
Subjects’ Ratings of Severity of Dysphonia	0.474	0.000*
Subjects’ Ratings Strain-Strangled	0.417	0.001*
Subjects’ Ratings Roughness	0.490	0.000*
Subjects’ Ratings Increased Expiratory Effort	0.275	0.039
Subjects’ Ratings Voice Tremor	0.539	0.000*
Subjects’ Ratings Voice Arrest	0.458	0.000*

^*^Findings was statistically significant at the .01 level

### Relationship between USDRS and DSQ

The similarity between the findings on the subjects’ DSQ and the clinicians’ USDRS was highly correlated for current severity of dysphonia, strain-strangled voice quality, roughness, voice arrest, and increased expiratory effort, but not for voice tremor (see Table 4).

**Table 4:** Correlation Between Subjects’ and Clinicians’ Ratings of Voice Symptoms *N* = 56

Voice Symptom	Pearson Correlation	Sig (2-tailed)
Dysphonia	0.353	0.008*
Strain-Strangled Voice Quality	0.389	0.003*
Roughness	0.466	0.000*
Voice Arrest	0.502	0.000*
Increased Expiratory Effort	0.454	0.000*
Voice Tremor	0.216	0.109

Note: All clinician ratings were made during review of the video recording of the Rainbow Passage and description of the Cookie Theft picture

^*^Findings were statistically significant at the .01 level

### Subject’s perception of changes in voice symptoms since original diagnosis

All subjects reported that at the time of their original diagnosis of AD-FLD their voice symptoms were worse than at the time of this study. This perceived reduction in symptom severity was statistically significant for all symptoms except roughness. See Table 5.

**Table 5:** Comparison of Severity of Current Symptoms with Severity at Diagnosis*

Vocal symptom N = 60	Corresponding DSQ Questions	Mean severity at initial diagnosis (SD)	Mean current severity (SD)	P
Severity of dysphonia	Q1, Q2	4.47 (0.68)	3.19 (0.89)	<0.00001*
Strain-Strangled Voice Quality (effortful phonation)	Q8, Q9	4.21 (0.86)	3.02 (1.00)	<0.00001*
Voice arrest (voice cuts off)	Q3, Q4	4.20 (0.97)	2.89 (1.02)	<0.00001*
Roughness (hoarse / husky)	Q5, Q7	3.41 (1.40)	3.01 (1.18)	0.10000
Voice tremor (shaking voice)	Q10, Q11	3.76 (1.31)	2.71 (1.19)	0.00002*
Increased expiratory effort	Q12, Q13	4.21 (0.90)	3.07 (1.11)	<0.00001*

Note: The DSQ symptom severity rating scale is a 5-point scale (i.e., 1 = mild, 2 = mild-to-moderate, 3 = moderate, 4 = moderate-to-severe, and 5 = severe). Q = question

^*^Findings were statistically significant at the .01 level

### Clinician ratings on the USDRS

Due to technical errors on four of the recordings, only 56/60 of the voice recordings were analyzed by four clinicians. Two speech-language pathologists and two laryngologists listened independently to the recording of each subject’s first paragraph of the Rainbow Passage (fifty-one words), description of the Cooke Theft Picture, and counting from eighty to eighty-nine. They then identified and rated the severity of the individual symptoms and overall severity of the dysphonia using the USDRS. The mean score for the severity of dysphonia was 4.2 (moderate impairment). The mean for roughness, strain-strangled, and increased expiratory effort was 4 indicating that these were the most remarkable symptoms. Symptoms of voice arrest, aphonia, and breathiness were mild with a mean of 1.

During a second evaluation of the recordings of the first paragraph of the Rainbow Passage, voice arrest was identified and timed by the four clinicians, and only 21/56 subjects, (38.1%) exhibited this symptom. Of those twenty-one individuals, 16 (28.6%) exhibited infrequent voice arrest (i.e., one or two instances of voice arrest), three (.05%) had occasional voice arrest (i.e., three to 10 occurrences of voice arrest), and just two (.03%) had more than 10 voice arrests. Eighteen (32%) of these subjects demonstrated momentary voice arrest (< 1 s), three (.05%) had brief voice arrest (1–2 s), but none had long voice arrest (> 2 s). Due to the infrequency of voice arrest, only descriptive statistics were performed.

### Inter-clinician reliability for rating vocal Symptomatology on USDRS

Four clinicians independently rated the severity of the dysphonia and the individual vocal symptoms on the USDRS for 56/60 subjects. Assessment of inter-clinician reliability for the clinician ratings was statistically significant, yielding a Cronbach Alpha of .905 for all parameters (see Table 6). The interclass correlations were significant for all symptoms on the USDRS except aphonia. See Tables 6 and 7. This high inter-clinician reliability may be associated with the clinician’s access to visual as well as audio information, which parallels the clinical assessment protocol.

**Table 6:** Inter-Clinician Reliability on the USDRS *N* = 56

	Cronbach’s Alpha	Interclass Correlation Coefficient F-Test
Reliability across all parameters on USDRS	0.905	<0.001*

^*^The reliability of clinician ratings of all perceptual symptoms was significant

**Table 7:** Inter-Clinician Reliability of Symptoms on the USDRS

Perceptual Rating	Interclass Correlation Coefficient	F Test Significance
Severity of Dysphonia	0.939	<0.001*
Roughness	0.840	<0.001*
Breathiness	0.444	0.003*
Strain-Strangled Quality	0.849	<0.001*
Abrupt Voice Initiation	0.755	<0.001*
Voice Arrest	0.413	0.007*
Aphonia	0.241	0.098
Voice Tremor	0.903	<0.001*
Increased Expiratory Effort	0.809	<0.001*
Speech Rate	0.694	<0.001*
Intelligibility	0.688	<0.001*

Note: All clinician ratings were made during review of the video recording of the Rainbow Passage and description of the Cookie Theft picture

^*^The reliability of clinician ratings of perceptual symptoms was statistically significant at the .01 level

## Discussion

The VHI-10 is frequently used to measure the impact of a voice problem on a patient’s quality of life. The present study asked whether the subjects’ perception of handicap as identified on the VHI-10 is correlated with the severity of voice symptoms associated with AD-FLD and selective medical history as identified on the DSQ (i.e., time since initial diagnosis, time since last injection, age at diagnosis, current age, total number of BoNT injections). The subjects’ perception of handicap as identified on the VHI-10 was positively correlated with the severity of the voice symptoms and negatively correlated with subjects’ age at the time of diagnosis. The strong positive correlation between the subjects’ ratings on the VHI-10 and the DSQ (e.g., dysphonia, strain-strangled, roughness, increased expiratory effort) suggests that the handicap associated with AD-FLD is related, not only to the severity of the dysphonia, but to the severity of the individual symptoms. Furthermore, subjects who were younger at the age of diagnosis (16–30 years) indicated greater handicap on the VHI-10 than those who were older when the symptoms began. This finding suggests that the psychosocial aspects of AD-FLD are not only relevant to the effectiveness of long-term management but require additional research to better understand their impact on quality of life.

Prerequisite to the effective management of patients with AD-FLD is agreement between the laryngologist and the speech-language pathologist with respect to the manifestations of the disease and its severity. Robust inter-clinician reliability ratings on the USDRS suggests that these clinicians are rating similar phenomena. Further, the strong correlation between the clinicians’ ratings on the USDRS and the subjects’ ratings on the DSQ suggests that clinicians and patients are identifying similar features and estimating the severity in an analogous way.

To date there has been little research investigating subjects’ perception of changes in their symptoms since the time of diagnosis. To that end, the current study asked subjects to compare the severity of their dysphonia as well as the individual characteristics at the time of the original diagnosis and then at the time of the study. All subjects rated the severity of their symptoms (with the exception of roughness) to be significantly more serious at the time of initial AD-FLD diagnosis than at the time of the study. Tremor and voice arrest were the two symptoms that were identified as having the greatest continuing improvement. At the time of diagnosis of AD-FLD, the mean severity of tremor and voice arrest was 4 on the DSQ (i.e., moderate-to-severe), but at the time of the study the mean rating on the DSQ was 2 (i.e., mild-to-moderate). This perceived reduction in severity raises questions as to whether the occurrence of voice arrest is potentially more responsive to treatment with BoNT than are other symptoms (e.g., roughness). Alternatively, do subjects develop adaptive behavioral strategies to minimize the frequency of voice arrest during connected speech, or is it the case that subjects overestimate the incidence of voice arrest at onset due to recall bias? If so, why was the severity of voice arrest and tremor overestimated more frequently than other symptoms (e.g., roughness) at the time of diagnosis?

Further research is necessary to explore whether voice arrest is more responsive to BoNT injections or behavioral control than other symptoms. Moreover, the improvement of AD-FLD symptoms since onset may result from an alteration in sensory feedback. The effect of BoNT injections on sensory pathways is an area of active research. It is hypothesized that BoNT alters sensory feedback in dystonia, resulting in improvement in symptoms long after the BoNT has dissipated. Individuals with oromandibular dystonia (OMD) and blepharospasm, for example, have noted improvement in their OMD symptoms following injection of BoNT into the orbicularis oculi alone. [24]

The VAM was used to identify voice arrests in first paragraph of the Rainbow Passage. Clinicians identified voice arrest in only 21/56 (38%) subjects. Several possible explanations may account for the infrequent occurrence of voice arrest in this subject population: voice arrest may not be a predominant characteristic of AD-FLD; voice arrest may continue to diminish following BoNT treatment; or subjects may learn to compensate for voice arrest more easily than for other symptoms. On the surface, it would be expected that clinicians familiar with spasmodic dysphonia would be as effective at documenting the incidence of voice arrest as with the occurrence of other voice symptoms. Further research is necessary to explore these alternative hypotheses.

## Conclusions

The diagnosis of AD-FLD is based on clinical symptoms and medical history. The high inter-clinician reliability of perceptual ratings by laryngologists and speech-language pathologists based on the USDRS, and the high correlation of clinician ratings with subjects’ ratings from the DSQ suggest that clinicians and subjects are rating similar phenomena using comparable criteria. The ratings of severity of dysphonia for both clinicians and subjects are related to the interaction of the symptoms and not to any single symptom. For subjects and clinicians alike, the most severe and frequently identified symptoms at the time of assessment were roughness, strain-strangled voice quality, and increased expiratory effort. Subjects and clinicians judged aphonia and voice tremor to be infrequent and less severe than other symptoms. The occurrence and duration of voice arrest as measured by the VAM were infrequent and relatively brief.

The severity of the handicap identified on the VHI-10 was strongly correlated, not only with greater severity of voice symptoms, but with a younger of age of onset of AD-FLD. These findings suggest that future research that addresses the psychosocial impact of AD-FLD in younger individuals may provide information that will enhance treatment and the quality of life for individuals suffering from adductor focal laryngeal dystonia.

## Additional files

Additional file 1: Disease Symptoms Questionnaire (DSQ). (DOCX 15 kb)

Additional file 2: Voice Arrest Measure (VAM). (DOCX 13 kb)

## Abbreviations

AD-FLD: Adductor focal laryngeal dystonia
BoNT: Botulinum toxin
DSQ: Disease Symptom Questionnaire
FLD: Focal laryngeal dystonia
USDRS: Unified Spasmodic Dysphonia Rating Scale
VAM: Voice Arrest Measure
VHI-10: Voice Handicap Index-10
