# Supplementary material for: Adductor focal laryngeal Dystonia: correlation between clinicians’ ratings and subjects’ perception of Dysphonia
Source: J Clin Mov Disord. 2017 Dec 13;4:20. doi: 10.1186/s40734-017-0066-y (PMC5727950; doi:10.1186/s40734-017-0066-y)
Supplement: Supplementary file 1 — Disease Symptoms Questionnaire (DSQ). (DOCX 15 kb) [file 40734_2017_66_MOESM1_ESM.docx]

**Disease Symptom Questionnaire (DSQ)**

**Subject ID Number: _______________**

1. How severe is your voice disorder today?

______________________________________________________________________________

Mild Mild-to-Moderate Moderate Moderate-to-severe Severe

1. How severe were your symptoms before you started receiving BoNT injections?

______________________________________________________________________________

Mild Mild-to-Moderate Moderate Moderate-to-severe Severe

1. Does your voice cut-off today (Voice Arrest)?

______________________________________________________________________________

Mild Mild-to-Moderate Moderate Moderate-to-severe Severe

1. Did your voice cut-off before you started BoNT injections (Voice Arrest)?

______________________________________________________________________________

Mild Mild-to-Moderate Moderate Moderate-to-severe Severe

1. Is your voice hoarse, rough, or husky today (Rough)?

______________________________________________________________________________

Mild Mild-to-Moderate Moderate Moderate-to-severe Severe

1. Compared with before you started receiving BoNT injections, how severe are your symptoms today?

______________________________________________________________________________

Less than before treatment The same as before treatment Worse

1. Was your voice hoarse, rough, or husky before you started BoNT injections (Rough)?

______________________________________________________________________________

Mild Mild-to-Moderate Moderate Moderate-to-severe Severe

1. Does your throat feel tight when you talk today (Strain-Strangled)?

______________________________________________________________________________

Mild Mild-to-Moderate Moderate Moderate-to-severe Severe

1. Did your throat feel tight when you were talking before you started BoNT injections (Strain-Strangled)?

______________________________________________________________________________

Mild Mild-to-Moderate Moderate Moderate-to-severe Severe

1. Does your voice shake when you talk today (Voice Tremor)?

______________________________________________________________________________

Mild Mild-to-Moderate Moderate Moderate-to-severe Severe

1. Did your voice shake before you started BoNT injections (Voice Tremor)?

______________________________________________________________________________

Mild Mild-to-Moderate Moderate Moderate-to-severe Severe

1. Do you push your voice and use effort when you talk today (Expiratory Effort)?

______________________________________________________________________________

Mild Mild-to-Moderate Moderate Moderate-to-severe Severe

1. Did you push your voice and use effort before you started BoNT injections (Expiratory Effort)?

______________________________________________________________________________

Mild Mild-to-Moderate Moderate Moderate-to-severe Severe

1. On a scale of 0 to 100, how do you rate your voice today?

Blitzer Current Percent of Normal Function Scale

0%-5%-10%-15%-20%-25%-30%-35%-40%-45%-50%-55%-60%-65%-70%-75%-80%-85%-90%-95%-100%

**Investigators Only**

Subject ID number: ____________

Time since initial diagnosis: _______years _______months

Time since last injection: _______ weeks

Age at diagnosis: _______years

Current age: _______years

Total number of BoNT treatments: __________

Other medical co-morbidities (including essential tremor, other dystonias, etc.):

Medications
