# Supplementary material for: Adductor focal laryngeal Dystonia: correlation between clinicians’ ratings and subjects’ perception of Dysphonia
Source: J Clin Mov Disord. 2017 Dec 13;4:20. doi: 10.1186/s40734-017-0066-y (PMC5727950; doi:10.1186/s40734-017-0066-y)
Supplement: Supplementary file 2 — Voice Arrest Measure (VAM). (DOCX 13 kb) [file 40734_2017_66_MOESM2_ESM.docx]

**Voice Arrest Measure (VAM)**

Subject Number _______________________

Rater________________________________ Date / / /

Review each subject’s recording of the first paragraph of the Rainbow Passage and indicate each occurrence of a voice arrest with a slash (/). Next to the /, indicate the duration of the break as “M” for momentary (less than 250 m.s.), “S” for short (less than 2 seconds), and “L” for long (longer than 2 seconds). Identify the type of breaks that occur.

Total Number of Breaks ______________________

Duration of Breaks = **Momentary** (less than 250 m.s.)

**Short** (less than 2 seconds)

**Long** (longer than 2 seconds)

Type of Breaks = Tight / Breathy

**THE RAINBOW PASSAGE**

**Fairbanks, G. (1960). *Voice and articulation drillbook* (2^nd^ ed.). New York: Harper & Row. p. 127.**

When the sunlight strikes raindrops in the air, they act like a prism and form a rainbow. The rainbow is a division of white light into many beautiful colors. These take the shape of a long round arch, with its path high above and its two ends apparently beyond the horizon. There is, according to legend, a boiling pot of gold at one end. People look, but no one ever finds it. When a man looks for something beyond his reach, his friends say he is looking for the pot of gold at the end of the rainbow.
